# Correction: Expression Patterns of Bovine CD1 *In Vivo* and Assessment of the Specificities of the Anti-Bovine CD1 Antibodies

**DOI:** 10.1371/journal.pone.0129755

**Published:** 2015-06-05

**Authors:** The PLOS ONE Staff

## Notice of Republication

This article was republished on May 13, 2015, to correct the third and seventh authors’ names. The third author’s given name is Chema and surname is El Messlaki. The seventh author’s given name is Ildiko and surname is Van Rhijn. The correct citation is: Nguyen TKA, Reinink P, El Messlaki C, Im JS, Ercan A, Porcelli SA, et al. (2015) Expression Patterns of Bovine CD1 *In Vivo* and Assessment of the Specificities of the Anti-Bovine CD1 Antibodies. PLoS ONE 10(3): e0121923. doi:10.1371/journal.pone.0121923

The publisher apologizes for the errors. Please download this article again to view the correct version. The originally published, uncorrected article and the republished, corrected article are provided here for reference.

## Supporting Information

S1 File Originally published, uncorrected article. (PDF)

S2 File Republished, corrected article. (PDF)
