# Supplementary material for: Correction: Expression Patterns of Bovine CD1 In Vivo and Assessment of the Specificities of the Anti-Bovine CD1 Antibodies
Source: PLoS One. 2015 Jun 5;10(6):e0129755. doi: 10.1371/journal.pone.0129755 (PMC4457881; doi:10.1371/journal.pone.0129755)
Supplement: S2 File — (PDF) [file pone.0129755.s002.pdf]

RESEARCH ARTICLE

Expression Patterns of Bovine CD1 *In Vivo* and Assessment of the Specificities of the Anti-Bovine CD1 Antibodies

Thi Kim Anh Nguyen[1¤], Peter Reinink[1], Chema El Messlaki[1], Jin S. Im[2], Altan Ercan[3], Steven A. Porcelli[4], Ildiko Van Rhijn[1,3]*

1 Department of Infectious Diseases and Immunology, Faculty of Veterinary Medicine, Utrecht University, 3584CL Utrecht, the Netherlands, **2** Section of Transplant Immunology, Department of Stem Cell Transplantation and Cellular Therapy, The University of Texas MD Anderson Cancer Center, Houston, United States of America, **3** Division of Rheumatology, Immunology, and Allergy, Brigham and Women's Hospital, Harvard Medical School, Boston, MA 02115, United States of America, **4** Department of Microbiology and Immunology, Albert Einstein College of Medicine, Bronx, NY 10461, United States of America

¤ Current address: Biotechnology Lab, R&D Center, Saigon Hi-Tech Park, Lot I3—Road N2, District 9, Ho Chi Minh City, Vietnam
* i.vanrhijn@uu.nl

CrossMark
click for updates

OPEN ACCESS

Citation: Nguyen TKA, Reinink P, El Messlaki C, Im JS, Ercan A, Porcelli SA, et al. (2015) Expression Patterns of Bovine CD1 *In Vivo* and Assessment of the Specificities of the Anti-Bovine CD1 Antibodies. PLoS ONE 10(3): e0121923. doi:10.1371/journal.pone.0121923

Academic Editor: Moriya Tsuji, Aaron Diamond AIDS Research Center with the Rockefeller University, UNITED STATES

Received: November 24, 2014

Accepted: February 8, 2015

Published: March 27, 2015

Copyright: © 2015 Nguyen et al. This is an open access article distributed under the terms of the Creative Commons Attribution License, which permits unrestricted use, distribution, and reproduction in any medium, provided the original author and source are credited.

Data Availability Statement: All relevant data are within the paper and its Supporting Information files.

Funding: This work was supported by Nederlands Wetenschappelijk Onderzoek, grant 824.02.002 and 836.08.001 to IVR. The funders had no role in study design, data collection and analysis, decision to publish, or preparation of the manuscript.

Competing Interests: The authors have declared that no competing interests exist.

Abstract

Research addressing the in vivo effects of T cell activation by lipids, glycolipids, and lipopeptides is hampered by the absence of a suitable animal model. Mice and rats do not express CD1a, CD1b, and CD1c molecules that present pathogen-derived lipid antigens in humans. In cattle, two *CD1A* and three *CD1B* genes are transcribed. The proteins encoded by these genes differ in their antigen binding domains and in their cytoplasmic tails, suggesting that they may traffic differently in the cell and thus have access to different antigens. In the current study, we describe the genomic organization of the bovine CD1 locus and transcription of bovine CD1 genes in freshly isolated dendritic cells and B cells from different tissues. After determining the specificity of previously only partly characterized anti-CD1 antibodies by testing recombinant single chain bovine CD1 proteins and CD1-transfected cells, we were able to determine cell surface protein expression on freshly isolated cells. Our study suggests that CD1b1 and CD1b3 are more broadly expressed than CD1b5, and CD1a2 is more broadly expressed than CD1a1. Pseudoafferent lymph dendritic cells express *CD1B* genes, but no transcription is detected in lymph nodes. Even though B cells transcribe *CD1B* genes, there is no evidence of protein expression at the cell surface. Thus, patterns of CD1 protein expression are largely conserved among species.

Introduction

Functionally and structurally, the CD1 family of proteins can be divided in two groups. Genes for group 1 CD1 proteins (CD1a, CD1b, and CD1c) are lacking in mice, but in humans, group

1 CD1 proteins are highly expressed on immature thymocytes and immature and mature dendritic cells (DCs). Group 2 CD1 (CD1d) molecules are expressed in humans and mice and have a much broader expression pattern. CD1d is present at a low level on many cell types, including B cells and non-hematopoietic cells. Minor cell populations with high levels of CD1d expression have been described, such as mantle zone B cells in the lymph node and marginal zone B cells in the spleen [1], and Ito cells in the liver [2]. CD1d presents antigen to invariant NKT cells, a cell population with a very limited and highly conserved T cell repertoire that can quickly release large amounts of cytokines. The strongest antigen for invariant NKT cells that is known is α-galactosylceramide.

Group 1 CD1 proteins are best known for their ability to present bacterial lipid antigens to T cells, though CD1a has been shown to be recognized by T cells without the addition of foreign antigen [3, 4]. Interestingly, mammalian species vary widely in the numbers of group 1 CD1 genes that are present in their genomes. For example, dogs have eight *CD1A* genes [5], guinea pigs have five *CD1B* genes and four *CD1C* genes [6], and humans have one gene for each isoform. Most of the known group 1 CD1-presented antigens are from mycobacterial origin, including *M*. *tuberculosis* and *M*. *leprae*, which cause human tuberculosis and leprosy, and *M*. *bovis*, which causes bovine tuberculosis. Identification of functional orthologs of CD1 molecules is relevant to the development and testing of experimental vaccines that incorporate lipid antigens.

Even though cellular expression patterns are relevant to in vivo function, humans are the only species in which the cellular expression pattern of group 1 CD1 molecules has been studied extensively. Less complete studies have been performed on rabbits [7], dogs [5], and guinea pigs [6]. Among the four guinea pig CD1b molecules, one seems to be differentially expressed on different types of dendritic cells [8]. Also, among the multiple canine CD1a molecules, two are expressed in skin and thymus, and one is expressed in thymus only.

Recently, we have used cattle (*Bos taurus*) as a model to study immune responses to CD1-presented mycobacterial lipid antigens [9, 10]. Among the bovine CD1 molecules, one functional *CD1A* gene, three functional *CD1B* genes (encoding CD1b1, CD1b3, and CD1b5), and one functional *CD1E* gene have been described [11]. Crystallographic studies of the bovine CD1b3 protein showed that it is able to bind lipid antigens in a way that is comparable to human CD1b, except that it can not bind ultra long carbon chain lipids [12]. Two *CD1D* genes that were previously thought to be pseudogenes were subsequently shown to be expressed at the cell surface, but can not bind α-galactosylceramide [13, 14]. Cattle are sensitive to natural infection with *M*. *bovis* and *M*. *avium* paratuberculosis, and we have shown that lipid antigens are recognized during these infections [15]. In addition, immunizations with glucose monomycolate, a glycolipid that was already known to be presented by human CD1b during leprosy and tuberculosis, showed that this compound is also immunogenic in cattle. In cattle, T cell recognition of glucose monomycolate could be blocked by the monoclonal antibody BCD1b.3, which is known to recognize human CD1b and bovine CD1b3, but whether it also recognizes other bovine CD1b molecules is unknown.

A considerable number of monoclonal antibodies was raised against ovine and bovine thymocytes and intestinal epithelial cells in the past, and their target molecule was suggested to be CD1 based on expression on thymocytes, immunoprecipitation of proteins with masses corresponding to a class I-like heavy chain and B2M, and in the case of the CC20 antibody, on cross-reactivity with human CD1b [16–24]. Since the description of the bovine CD1 family, only the CD1a, CD1b3, and CD1d1 transcripts were cloned and the recognition by some existing monoclonal antibodies of these two proteins was demonstrated [11, 13]. Here we determine the specificity of the monoclonal antibodies CC14, CC20, CC122, SBU-T6 (also called 20.27), BCD1b.3, and CC43 using recombinant proteins in ELISA assays and transfected cell lines in

FACS analyses, which enabled us to interpret ex vivo antibody staining patterns observed with these antibodies. In addition, using gene-specific PCR, we describe the differential transcription of bovine CD1b1, CD1b3, CD1b5, CD1d, and CD1a, and a newly discovered bovine CD1a molecule, which we named CD1a2, on sorted DCs, B cells, and in vitro cultured DCs.

Materials and Methods

Animals, tissue samples, and cDNA synthesis

For flow cytometry and nucleic acid extractions, bovine tissue samples (prescapular lymph node, thymus, peripheral blood drawn from the jugular vein, and pseudoafferent lymph) were collected from a Holstein-Frisian bull of three months of age with no clinical signs of disease. The experiments were carried out in the Netherlands and were approved by the Animal Ethical Committee of the University Utrecht, 0409–0801. The procedure for obtaining pseudoafferent lymph has been described previously [25]. PBMC were prepared using a standard Ficoll density gradient. Single cell suspensions of bovine tissues were used without further purification. RNA isolation and cDNA synthesis was performed on $2^{*}10^{4}$ sorted cells. RNA was isolated with the Qiagen RNAEasy kit, followed by first strand cDNA synthesis with Multiscribe reverse transcriptase.

Molecular cloning of bovine CD1 transcripts

Full length CD1b3 had been cloned previously [11]. Bovine CD1b1 and CD1a2 were identified as ESTs (EH164202 and BC149754, respectively) based on a BLAST search with the predicted CD1b1 and CD1a2 transcript and confirmed by sequencing (IMAGE clones 8452304 and 8436847 respectively, from Geneservice UK). No full length CD1b5 transcript was present in the databases. Based on the genomic sequence of CD1b5, primers for CD1b5 were designed:

CD1b5FullLengthFor: 5′ -AGTTCTACTTCCCATTGAAATGCTGCTTCTG-3′ ; CD1b5FullLengthRev: 5′ -GTAATTGCTCTAAATGGGAAAGAAGACACG-3′ , as described previously [12]. PCR was performed with PFU Turbo polymerase (Stratagene) according to the protocol of the manufacturer under the following cycling conditions: an initial denaturation of 7 min. at 95°C, followed by 35 cycles of 30 sec. at 95°C, 45 sec. at 58°C, 1 min. at 72°C, followed by a final elongation step of 7 min. at 72°C. PCR products were cut from an agarose gel, purified, and ligated in a Topo4blunt vector for sequencing and in pcDNA3.1 for expression.

Monoclonal antibodies, FACS analyses, and cell sorting

BoMac cells were transiently transfected with cloned full length wild type CD1b1, CD1b3, and CD1b5 in pcDNA3.1 using Fugene-6 reagent (Roche) according to the manufacturer's protocol, and analysed 48 hours after transfection. K562 cells transfected with bovine CD1d and human CD1a, and C1R cells transfected with bovine CD1b and CD1a were described previously [3, 12]. The anti bovine CD1 antibodies CC20 (IgG2a), CC43 (IgG2b), and CC122 (IgG1) were kindly provided by Dr. C. J. Howard, Compton, UK; 20.27 SBU-T6 (IgG1) was obtained from the European Collection of Cell Cultures; CC14 (IgG1) and CC118 (IgG1) were kindly provided by Dr. J. C. Hope, Compton, UK; BCD1b.3 (IgG1) was provided by Dr. D. B. Moody, Brigham and Women's Hospital, Boston, goat anti mouse FITC and goat anti mouse PE were obtained from Becton Dickinson. PE-labelled anti bovine Ig light chain antibody IL-A59 was used as B cell marker (Serotec).

DC culture

DC were cultured as described [26]. In short, PBMC were prepared using Histopaque ficoll (Sigma-Aldrich). The CD14+ cells were positively selected with anti human CD14 MicroBeads (Miltenyi Biotec) on a MACS separator. The CD14+ cells were then cultured in 24-well plates at a density of 10^6 cells/ml in RPMI/10%FCS supplemented with bovine IL-4 and bovine GM-CSF. Immature DC cells were analyzed by flowcytometry and CD1 gene-specific PCR.

Gene-specific PCR

To be able to confirm transcription of *CD1A1*, *CD1A2*, *CD1B1*, *CD1B3*, *CD1B5*, and *CD1D*, we designed the following gene-specific primers:
CD1a1For: 5' CCATTTCCTTCAAAGTCATCTGTGTCC 3′ ;
CD1a1Rev: 5' AAGAGGAAATGTGGGCAGGTATCAC 3′ ;
CD1a2For: 5' CTCATCATTTTATAGCCAATTCTTTGC 3′ ;
CD1a2Rev: 5' AAGAGGAAATGTGGGCAGGTATCAC 3′ ;
CD1b1For: 5'TTCACTTGCGGAATGCAGGATCGA 3′ ;
CD1b1Rev: 5' GCACAAAACTTCTGCCCCCTGAA 3′ ;
CD1b3For: 5' TGATGATGAGGTGACTGAGCTGGT 3′ ;
CD1b3Rev: 5' GCTGTCTGGTGCAGGCACACAA 3′ ;
CD1b5For: 5' TTCAGTGATGAGGAGGTGGCTGAGA 3′ ;
CD1b5Rev: 5' AGTAATGAATACACAAAACCACTGTGCATCA 3′ ;
CD1dFor: 5' GCAGCATACACTTCTGGTTTATCG 3′ ;
CD1dRev: 5' CAGGCCCAGGACTGGGGCCACTGG 3′ .
The *CD1D* genes that are present in cattle are highly homologous and our CD1d primer set was designed to cover them all (S1 Fig.). All forward primers were located in the α1 domains and the reverse primers in the α2 domains, except for the CD1dRev primer, which was located in the α3 domain. The specificity of these gene-specific PCRs was confirmed by using vectors containing full length cloned CD1b1, CD1b3, CD1b5, and CD1a as a template for each of these gene specific PCRs. Using the same PCR conditions as described for the cloning of CD1b5, but with an annealing temperature of 70°C for CD1b1, CD1b3, and CD1b5, and 62°C for CD1a, PCR products could only be detected using the vector DNA with the cloned gene for which the primer set was designed as a template, and not using the other CD1 genes as a template.

Recombinant CD1 protein design and expression

Single chain bovine CD1b1, CD1b3, and CD1b5 constructs were generated as described previously for human CD1d [27] by linking the sequence for the entire bovine ß-2-microglobulin (B2M) at the N-terminal sequence of the α1 domain of the CD1 proteins by the insertion of a sequence encoding a flexible peptide linker (GGGSGGSGSGGGA). Sequence elements encoding the BirA enzymatic biotinylation site (HVGLNDIFEAQKIEWHEGH), and a hexahistidine (HHHHHH) tag were added to the C terminus of the α3 domain of the CD1 proteins. In addition, we used the CD1b5 splice variant lacking part of the α3 domain. The amino acid sequences of the fusion proteins are shown in S2 Fig. The supernatants of HEK 293T cells transiently transfected with these constructs were collected and purified on Ni-NTA columns. The size and concentration of the recombinants proteins were determined using SDS-PAGE and Coomassie blue staining.

PNGase-F treatment and ELISA

All ELISAs were performed on Costar high bind 96-well plates. For ELISA, coating of single chain recombinant proteins (0.5 μg/well) was followed by washing, blocking with PBS/1%BSA/

0.05% tween 20 (Merck), incubation with detecting antibody, washing, incubation with polyclonal rabbit anti mouse IgG-HRP (Roche), washes with PBS/0.05% tween and PBS, and incubation with ABTS solution (Roche). ELISA plates were read at 405 nm. Bovine single chain CD1b3 was deglycosylated using the PNGaseF kit from New England Biolabs. The native deglycosylation reaction was performed in G7 reaction buffer for five days at 37°C for 5 days. For mock treated protein, PNGase was omitted, but otherwise treated equally. The same samples that were used for ELISA were analyzed on a denaturing 14% SDS-page gel followed by Coomassie blue staining, together with samples that were deglycosylated in glycoprotein denaturing buffer, NP40 buffer, and G7 reaction as a control for maximum deglycosylation obtained under denaturing conditions.

Results

Identification of two new CD1A genes and comparison of transcripts of multiple bovine CD1B and CD1A genes

Previous descriptions of the bovine family of CD1 genes were all based on incompletely assembled genomic data [11, 13]. In the Btau_4.0 release of the bovine genome, the CD1 locus contained big gaps, and some CD1 genes were found on "chromosome fragments". Here we studied the UMD3.1 assembly which contains a fully assembled, though incompletely annotated CD1 locus and found a novel *CD1A* gene and two more *CD1D* genes that are extremely homologous to the previously described *CD1D1* and *CD1D2* (Fig. 1A). We called the newly identified *CD1A* gene *CD1A2*, and the gene that was previously named *CD1A* was renamed *CD1A1*. Like the human locus, the boundaries of the bovine CD1 locus are formed by genes encoding olfactory receptors and KIRREL.

Cloning of full length CD1b5 (GU325785) was described earlier [12]. Here we describe a CD1b5 splice variant that lacks part of the α3 domain (JN033695). This splice variant was also present in the EST database (EH160235), but sequencing the corresponding IMAGE clone 8444381 (Geneservice UK) showed that the insert corresponded to an incomplete transcript. Fig. 1B shows an alignment of the predicted amino acid sequences of the full length bovine CD1b transcripts and the CD1b5 splice variant, (upper panel), and the two full length bovine CD1a transcripts (lower panel). Note that the cytoplasmic tails of CD1b3 and CD1b5 are very similar to each other, with a YXXZ motif and a dileucine motif, but the cytoplasmic tail of CD1b1 is very different and does not seem to contain any known motifs. The cytoplasmic tails of CD1a1 and CD1a2 are very different from each other and from the cytoplasmic tail of human CD1a, and do not contain any known motifs.

Characterization of the specificity of monoclonal antibodies using recombinant proteins and transfectant cells

Full length cloned CD1b1, CD1b3, and CD1d transcripts were used to transiently transfect the bovine macrophage cell line BoMac (Fig. 2A). Mock transfected and transfected cells were stained with the unlabeled monoclonal antibodies CC14, CC20, CC43, CC122, SBU-T6, and BCD1b.3, followed by goat anti mouse-PE. CD1b3 was recognized by all of these antibodies except CC43. CD1b1 was recognized by SBU-T6 and BCD1b.3. CD1d was recognized by CC43 and SBU-T6. Transfections of BoMac cells with CD1b5 did not lead to any detectable antibody staining (data not shown). However, it was not clear whether this was caused by inefficient transfection and/or translation or lack of recognition by antibodies. Also, we did not detect any intracellular staining of CD1b5.

A

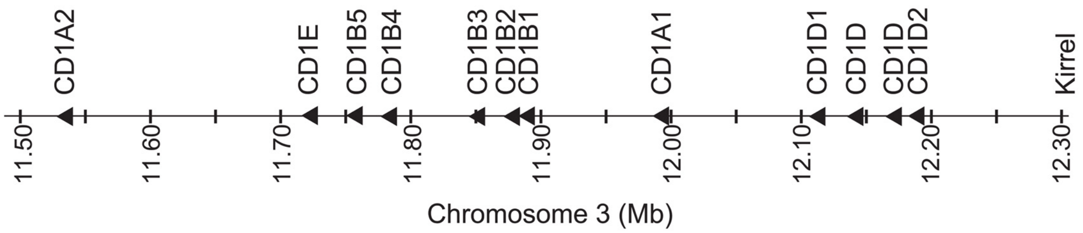

B

CD1b transcripts

```
                Leader       |                                                           α1 domain
CD1b3    MLLLPFLLLGVILPGGDNEDVFQGPTSFHLMQISTFVNSTWAQNQGSGWLDDLQIHGWESDSGTAIFLKPWSKGNFSDDEVTELVDLFRAYFIGFTREVQ
CD1b1    -----L------------A-------------------N-C----------------L---L----------------------Q------L----CGM-
CD1b5    -----L---A--V-------A---------I-----A----T----------------D-------------------E--A-MEE---V------L---
CD1b5    -----L---A--V-------A---------I-----A----T----------------D-------------------E--A-MEE---V------L---
splice var.

                    |                                 α2 domain
CD1b3    DRVNEFQLEYPFVIQVTAGCELHSGEAIESSLRGALGGLDFVSIQNHSCVPAPDSGSRGQKFCALTTQYQGISDIIERLLSETCPRYLLGVLDAGKAELQ
CD1b1    ---------------L------------G---------------------------F--------M--NAV-- Y----------Q--------------
CD1b5    -I-S---F-------GI--------KV-Q-F--AGF-----M--K-R----E-EG--DA-W--VFI------LR--DT----------------------
CD1b5    -I-S---F-------GIT-------KV-Q-F--AGF-----M--K-R----E-EG--DA-W--VFI------LR--DT----------------------
splice var.

               |                                      α3 domain
CD1b3    RQVKPEAWLSSGPTPGPGRLLLVCHVSGFYPKPVRVMWMRGEQEQPGTQQGDLMPNADWTWYLRVTLNVAAGEAAGLNCRVKHSSLGDQDIILYWGHPTS
CD1b1    ----------------------------------------------------I-----------------------------------------------
CD1b5    ---------------R---------------------------------NI-------------D---------S--------------------KY
CD1b5    ---------------R---------------------------------NI--------TPQVHWLDIC-NNSA-LDPFDM-CIMALEALVISDYLV-
splice var.

            |       TM and CT
CD1b3    IGLILVAIIIPSLILLICLALWFWRRWSYQNIL
CD1b1    ---------V----------FGVL-FMGSHRVGHD
CD1b5    ---------V-----------L-------T--
CD1b5    PRRVFFP-
splice var.
```

CD1a transcripts

```
                Leader       |                                                           α1 domain
CD1a1    MLFLQLPLLLALLVGGDNEQNKNSVPEGFQEPISFEVICVLSFHNSSWVQSLGSGWLGELQTHGWKSNSGTFIYIWPWSKGNFSNEELMELQNYLHTSFV
CD1a2    -----IA---S--PC--S-ND       -----T-KI-RIS--YSQFFA-N---A--D-----A-DN--DRV---R------------DVE-V---F-I

                                                                                  α2 domain
CD1a1    RFLQAFYSHARKWQFEYPFEVQIAKGCELHAGEVPVGFMRIAYQGSDFLSFQNKSWVSSPEGGKRAQVLRRLFNLFRGGQEIIHKLLSDTCPRFLLGLLD
CD1a2    -LG-VLHN--SQ--LQ----L---G---M-IRDAS---V--G---------KDV--------IS--FVCT----Y--T--------------------

                |                                                             α3 domain
CD1a1    AGKAYLQRQVRPEAWLSLGPSPGPGQLTLVCHISGFYPKPIWVMWMRGEQEQQGTQRSDVLPNPDGTSYLRVSLDVEASEASGLSCRVRHSSLGGQDIIL
CD1a2    ----------------P---------M----V------------------------------A---W--------------------------------

               |       TM and CT
CD1a1    YWDHHSSVGWIALAVIVTLVLMAGLAFWLWKHWTHRESPSSVLPLE
CD1a2    -------TA--I--M--P---L-------R-S-STYM-DA
```

Fig 1. CD1 locus and sequences of multiple bovine CD1b and CD1a molecules. A The bovine CD1 locus based on the UMD3.1 assembly. The genes were named based on sequence alignment with previously published names [11, 13]. We called the newly identified *CD1A* gene *CD1A2*, and the gene that was previously named *CD1A* was renamed *CD1A1*. B Bovine CD1b and CD1a cDNA sequences were translated and aligned. The accession numbers of the sequences included in this figure are: BoCD1b3: DQ192542; BoCD1b1: EH164202; BoCD1b5 splice variant: JN033695; BoCD1b5: GU325785; BoCD1a(1): DQ192541; BoCD1a2: BC149754. TM: Transmembrane part; CT: Cytoplasmatic tail;—identical residue.

doi:10.1371/journal.pone.0121923.g001

As an alternative approach, we produced His-tagged fusion proteins of bovine ß2M and several CD1 molecules. The cloned and sequenced full length CD1b1, CD1b3, and CD1b5 transcripts, and the CD1b5 splice variant were used as starting material for the cloning and production of soluble B2M-CD1 single chain proteins (S2 Fig.). ELISA plates were coated with

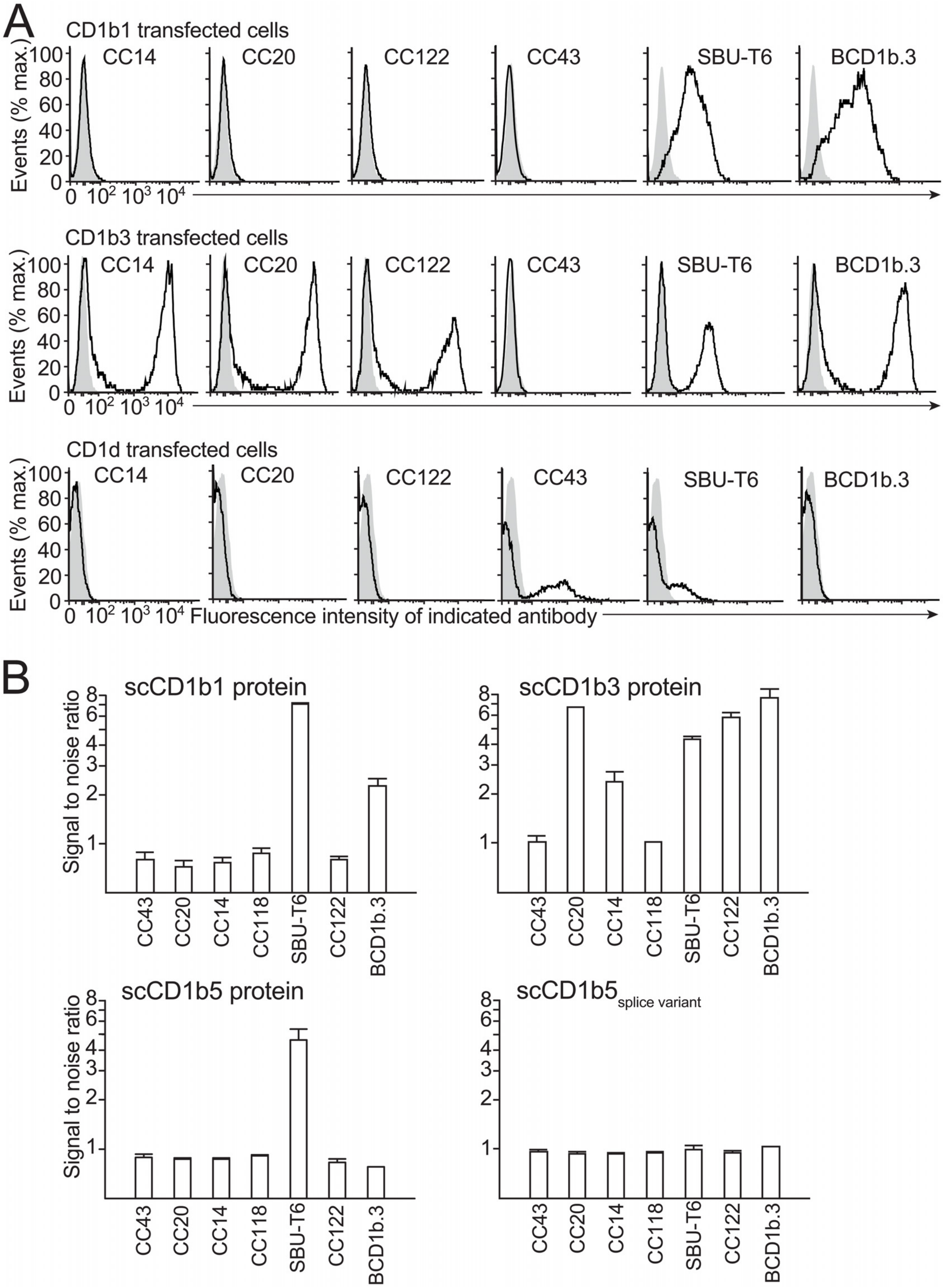

Fig 2. Antibody recognition of transfected cells and recombinant proteins. Bomac cells were transiently transfected with bovine CD1b1, CD1b3, and CD1d and stained with the monoclonal antibodies CC14, CC20, CC43, CC118, CC122, SBU-T6, and BCD1b.3 (black lines), or isotype control antibody (grey area) (A). Single chain recombinant proteins CD1b1, CD1b3, CD1b5, and splice variant of CD1b5, were coated on ELISA plates and tested for reactivity with the monoclonal antibodies CC14, CC20, CC43, CC118, CC122, SBU-T6, and BCD1b.3. The signal to noise ratio is defined the means of the duplicate OD values obtained from wells coated with single chain CD1 proteins divided by the means of the OD values obtained by wells without single chain CD1 protein (B). Error bars: standard deviation of triplicate measurements.

doi:10.1371/journal.pone.0121923.g002

the recombinant proteins and recognition of the proteins by the aforementioned panel of monoclonal antibodies was tested (Fig. 2B). Recombinant CD1b1 protein was confirmed to be recognized by SBU-T6 and BCD1b.3, but not by the other antibodies; recombinant CD1b3 protein was again recognized by all antibodies except CC43, recombinant CD1b5 protein was recognized by SBU-T6 only, and the naturally occurring splice variant was not recognized by any of the antibodies.

CD1 gene expression by B cells and DCs

Because the considerable differences among the antigen binding domains and cytoplasmic tails of the bovine CD1 molecules have implications for their antigen capture and presenting capacities, we set out to determine which CD1 genes are transcribed by professional APCs in cattle. In addition, these data will clarify whether cellular expression patterns are conserved among species. Long before it was known that cattle do not have a *CD1C* gene, it was suggested that the SBU-T6 antibody might recognize the bovine and ovine homolog of CD1c [28]. The antibody SBU-T6 has later been characterized as a "pan-CD1" antibody, recognizing most or all bovine and ovine CD1 molecules. Confirming its pan-CD1 specificity, it has previously been shown to recognize boCD1b3 and boCD1a, the only boCD1 molecules cloned at that time [11], it has been used to immunoprecipitate ovine CD1e [29], and here we describe its recognition of boCD1d, boCD1b1 and boCD1b5 (Fig. 2). In fact, SBU-T6 also recognizes a canine CD1a and CD1b molecule [5], and two guinea pig CD1b molecules [8]. To check for CD1 gene transcription by bovine B cells and DCs, we sorted SBU-T6 expressing cells from fresh PBMC, pseudoafferent lymph, and lymph node (Fig. 3 and S3 Fig.). Cells that were double positive for SBU-T6 and IL-A59 (Ig light chain), were considered to be CD1-expressing B cells, cells that were IL-A59 positive and SBU-T6 negative were considered to be CD1-negative B cells, and cells that were SBU-T6 positive and IL-A59 negative were considered CD1-expressing DCs (Fig. 3A).

Gene specific PCR primers were designed and validated on cloned CD1b1, CD1b3, CD1b5, CD1a1, CD1a2, and CD1d transcripts. The primers were confirmed to amplify one gene of one isoform, and do not cross react with other CD1 genes. Gene-specific PCRs on cDNA synthesized from RNA from sorted APC populations showed that CD1b1 and CD1b3 had a much broader expression pattern than CD1b5, which was only found in DCs sorted from pseudoafferent lymph (Fig. 3B). CD1b was not transcribed by lymph node-derived cells. CD1a1 transcript was only present in DCs, but CD1a2 had a broader expression pattern, including lymph node B cells. CD1d had a broad expression pattern. In addition to these ex vivo cells, in vitro cultured DCs were characterized by FACS and PCR (Fig. 3C, left). Even though not all cells in these cultures expressed CD1 (Fig. 3C, right), all CD1 genes were transcribed in the cultures, except CD1B5.

The SBU-T6 epitope is dependent on glycosylation

We have recently reported the human cell line K562 transfected with bovine CD1d [13]. That cell line is recognized by CC43 [13], but interestingly not by SBU-T6. Here we report that the BoMac bovine macrophage cell line transfected with bovine CD1d is recognized by CC43 and SBU-T6. One obvious difference between the experiments is the species from which the cell line was derived. Initially we hypothesized that the epitope for SBU-T6 is formed by bovine CD1 in complex with bovine ß2M, but not in complex with human ß2M. An important argument against this hypothesis was the observation that SBU-T6 recognizes huCD1a on K562 cells, but not on C1R cells, which are both human cell lines (data not shown).

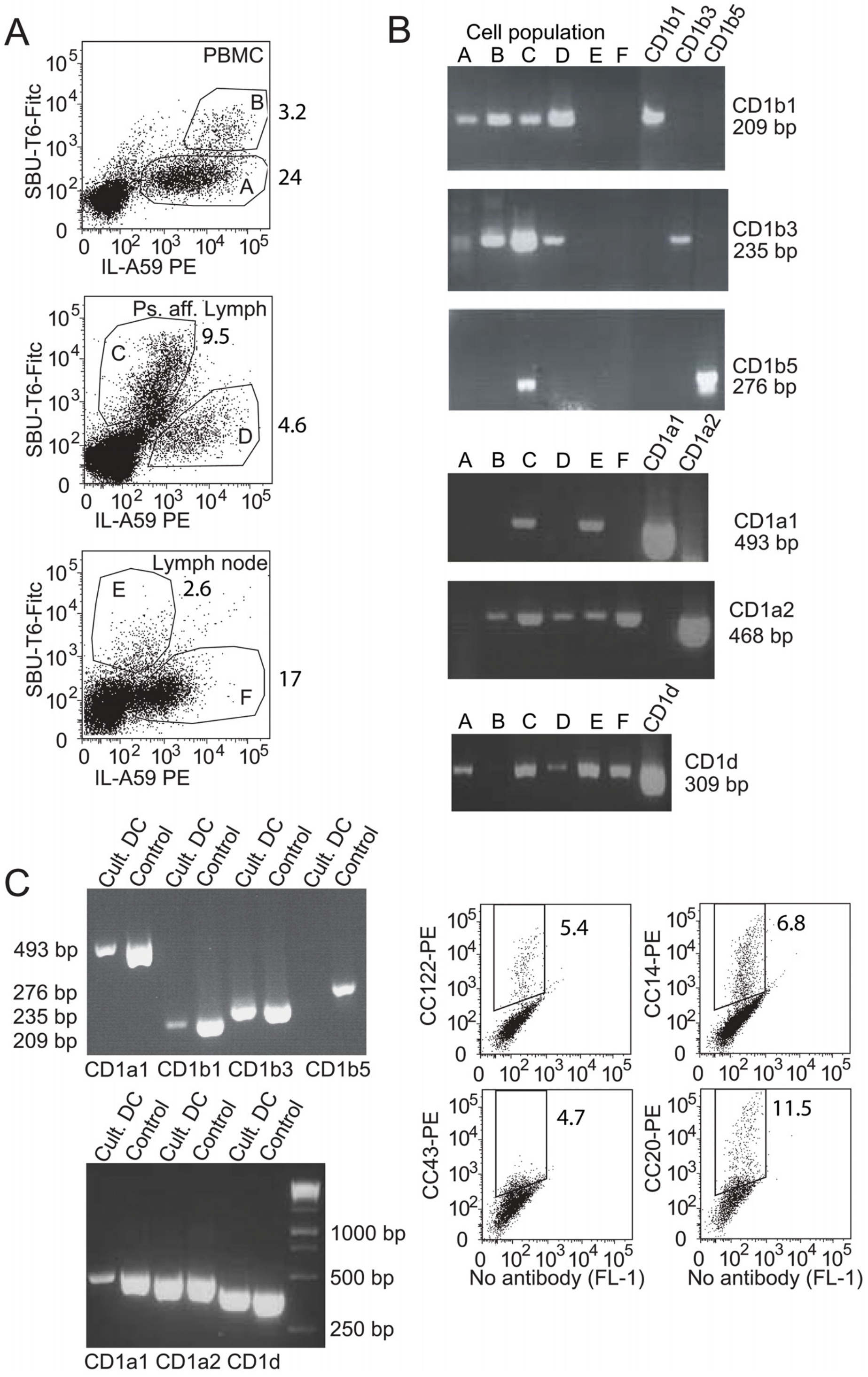

Fig 3. Transcription of individual CD1 genes by bovine APCs. A Cells from PBMC (top panel), pseudoafferent lymph (middle panel), and prescapular lymph node (lower panel) were sorted based on expression of the Ig light chain (IL-A59), and CD1 (SBU-T6). The cDNA synthesized from the sorted cell populations was subjected to CD1 gene-specific PCR (B). C The CD1 expression of immature DCs, derived in vitro by treatment of monocytes with GM-CSF and IL-4, was confirmed and cDNA derived from the DCs, as well as cloned transcript as positive control template (Control), was subjected to CD1 gene-specific PCR.

doi:10.1371/journal.pone.0121923.g003

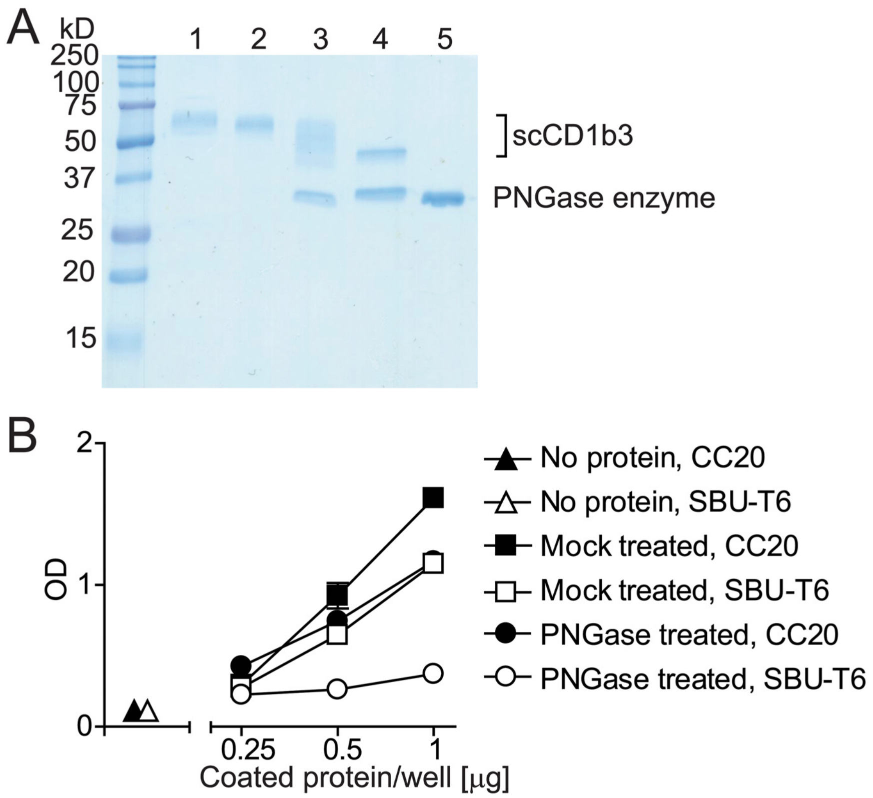

Fig 4. The influence of deglycosylation on CD1 recognition by SBU-T6. Single chain bovine CD1b3 was treated with PNGase F and analysed by SDS-Page followed by Coomassie stain (A). Lane 1: untreated protein; Lane 2: mock treated protein; Lane 3: PNGase F treated protein, non denaturing conditions during PNGase F treatment; Lane 4: PNGase F treated protein, denaturing conditions during PNGase F treatment; Lane 5: PNGase F enzyme only. The non denatured PNGase F treated protein and mock treated protein were coated on an ELISA plate and analysed for the reactivity with SBU-T6 and CC20 (B).

doi:10.1371/journal.pone.0121923.g004

An alternative hypothesis, inspired by the fact that CD1 molecules are known to be heavily glycosylated, was that the recognition of CD1 by SBU-T6 is influenced by the glycosylation status of the molecules. To test this we digested single chain CD1b3 under native conditions with PNGase F or mock treated it and tested its recognition by SBU-T6 and CC20 (Fig. 4). Even though SDS-gel followed by Coomassie staining showed that the protein was incompletely deglycosylated (Fig. 4A), the ELISA signal using SBU-T6 as a detecting antibody was greatly reduced, while the ELISA signal using CC20 as a detecting antibody was barely affected (Fig. 4B). From this we conclude that N-glycosylation of CD1b3 is required for recognition by SBU-T6.

Discussion

The widely variable numbers of CD1 genes among mammals is puzzling. We have previously shown that bovine group 2 CD1 (CD1d) is expressed, but does not seem to fulfill the model function of presenting α-galactosylceramide to NKT cells and cause the release of cytokines [13]. One question we had is whether group 1 and group 2 CD1 molecules, even when the numbers of genes, and possibly their functions are different, follow the typical group 1 and group 2 CD1 expression patterns in the sense that group 1 CD1 is present on thymocytes and dendritic cells, and not on non-hematopoietic cells, while group 2 CD1 has a broader expression pattern

and includes B cells and non-hematopoietic cells. Another question that we asked was whether the genes that belong to one CD1 isoform are differentially expressed in cattle.

We found that the bovine CD1 genes that belong to one CD1 isoform are differentially expressed while the general expression pattern of group 1 and group 2 CD1 in cattle is conserved. Bovine B cells transcribe CD1d, and bind to CC43 and SBU-T6, so we conclude that the CD1d transcript is translated into CD1d protein on B cells. Also, before it was known what molecule they recognize, CC43 and CC118, two antibodies with indistinguishable specificity, were known to recognize bovine thymocytes and intestinal epithelial cells [17], which is consistent with the typical group 2 CD1 expression pattern. Unlike in humans, we have shown here that CD1b1 and CD1b3 are transcribed by B cells but B cells are known to not bind the CD1b specific antibodies CC14, CC20 [17, 18], and BCD1b.3 (Van Rhijn, unpublished), so it appears that the CD1b genes are transcribed but not translated into protein. CD1a2, again, unlike in humans, is transcribed by bovine B cells. We did not have an antibody that distinguishes CD1a2 protein from CD1d protein on B cells, so we do not know whether bovine CD1a2 protein is present on B cells. Regardless, the expression of CD1d on B cells is a conserved feature. CD1c is also expressed by human B cells, but cattle do not have a gene for CD1c. Remarkably, bovine B cells transcribe CD1b1, CD1b3, and CD1a2, which human B cells do not.

We have shown here that CD1b transcription by DCs in lymph nodes is not detectable while CD1b transcription in pseudoafferent lymph cells is high. This latter finding is in agreement with CD1b protein expression on (pseudo)afferent lymph DCs as reported by us and others [25, 26, 28], and by the inability of lymph node cells to present the CD1b-presented antigen glucose monomycolate to T cells as compared to PBMC, which do present glucose monomycolate to T cells [9]. From this we conclude that pseudoafferent DCs strongly downregulate their CD1b expression upon entry into the lymph node.

SBU-T6 is an antibody that recognizes many CD1 isoforms but not under all circumstances. The antibody SBU-T6 recognizes almost all bovine and ovine CD1 molecules, a canine CD1a and CD1b molecule [5], and two guinea pig CD1b molecules [8]. Interestingly, the human cell lines K562 transfected with bovine CD1d and C1R with bovine CD1b3 are not recognized by SBU-T6 while BoMac cells transfected with these molecules are. We have found a strong effect of deglycosylation of CD1 on recognition by SBU-T6. It is possible that the epitope of SBU-T6 is a conformational epitope formed by CD1b protein and its glycosylation. Knowing this does not affect the interpretations and conclusions of the work presented here, but it has implications for interpretation of negative staining, especially of transfected cells.

Finally, our CD1 transcription data and the data that definitively confirmed the specificity of the monoclonal antibodies CC14, CC20, CC43, CC122, and BCD1b.3 allow us to fine-tune the interpretations of previously published histological and flow cytometric studies using these antibodies [16, 20, 21, 28, 30]. Our studies also confirm the general conclusion that group 2 CD1 (CD1d) expression patterns are conserved among species, despite the fact that the function of bovine CD1d has not been confirmed to be equivalent to the function of human and murine CD1d, and that group 1 CD1 molecules are differentially expressed among professional APCs, and that there might be a discrepancy between group 1 CD1 transcription and protein expression.

Supporting Information

S1 Fig. Aligment of the genomic sequences of the four bovine CD1D genes. The α1, α2, and α3 domains are in bold, and the location of the CD1dFor and CD1dRev sequences is marked by a cyan box.
(EPS)

S2 Fig. Alignment of single chain CD1 proteins used in this study.
(EPS)

S3 Fig. Analysis of sorted cells. Directly after FACS-sorting cells in the indicated gates A-F, a small aliquot of populations A and C-F was analyzed using the same instrument, but not collected.
(EPS)

Author Contributions

Conceived and designed the experiments: TKAN PR CEM AE IVR JSI SAP. Performed the experiments: TKAN PR CEM. Analyzed the data: TKAN PR CEM IVR. Contributed reagents/materials/analysis tools: IVR JSI SAP. Wrote the paper: TKAN PR CEM AE IVR.

References

1. Dougan SK, Kaser A, Blumberg RS. CD1 expression on antigen-presenting cells. Curr Top Microbiol Immunol. 2007; 314:113–41. PMID: 17593659
2. Winau F, Hegasy G, Weiskirchen R, Weber S, Cassan C, Sieling PA, et al. Ito cells are liver-resident antigen-presenting cells for activating T cell responses. Immunity. 2007; 26(1):117–29. PMID: 17239632
3. de Jong A, Pena-Cruz V, Cheng TY, Clark RA, Van Rhijn I, Moody DB. CD1a-autoreactive T cells are a normal component of the human alphabeta T cell repertoire. Nat Immunol. 2010; 11(12):1102–9. doi: 10.1038/ni.1956 PMID: 21037579
4. de Jong A, Cheng TY, Huang S, Gras S, Birkinshaw RW, Kasmar AG, et al. CD1a-autoreactive T cells recognize natural skin oils that function as headless antigens. Nat Immunol. 2014; 15(2):177–85. Epub 2013/12/24. doi: 10.1038/ni.2790 PMID: 24362891
5. Looringh van Beeck FA, Zajonc DM, Moore PF, Schlotter YM, Broere F, Rutten VP, et al. Two canine CD1a proteins are differentially expressed in skin. Immunogenetics. 2008; 60(6):315–24. doi: 10.1007/s00251-008-0297-z PMID: 18488214
6. Dascher CC, Hiromatsu K, Naylor JW, Brauer PP, Brown KA, Storey JR, et al. Conservation of a CD1 multigene family in the guinea pig. J Immunol. 1999; 163(10):5478–88. PMID: 10553074
7. Hayes SM, Knight KL. Group 1 CD1 genes in rabbit. J Immunol. 2001; 166(1):403–10. PMID: 11123318
8. Hiromatsu K, Dascher CC, LeClair KP, Sugita M, Furlong ST, Brenner MB, et al. Induction of CD1-restricted immune responses in guinea pigs by immunization with mycobacterial lipid antigens. J Immunol. 2002; 169(1):330–9. PMID: 12077262
9. Nguyen TK, Koets AP, Santema WJ, van Eden W, Rutten VP, Van Rhijn I. The mycobacterial glycolipid glucose monomycolate induces a memory T cell response comparable to a model protein antigen and no B cell response upon experimental vaccination of cattle. Vaccine. 2009; 27(35):4818–25. doi: 10.1016/j.vaccine.2009.05.078 PMID: 19538998
10. Nguyen TK, Wieland W, Santema W, Hoeboer J, van Eden W, Rutten V, et al. Immune response of cattle immunized with a conjugate of the glycolipid glucose monomycolate and protein. Vet Immunol Immunopathol. 2011; 142(3–4):265–70. doi: 10.1016/j.vetimm.2011.05.024 PMID: 21684019
11. Van Rhijn I, Koets AP, Im JS, Piebes D, Reddington F, Besra GS, et al. The bovine CD1 family contains group 1 CD1 proteins, but no functional CD1d. J Immunol. 2006; 176(8):4888–93. PMID: 16585584
12. Girardi E, Wang J, Mac TT, Versluis C, Bhowruth V, Besra G, et al. Crystal structure of bovine CD1b3 with endogenously bound ligands. J Immunol. 2010; 185(1):376–86. doi: 10.4049/jimmunol.1000042 PMID: 20519644
13. Nguyen TK, Koets AP, Vordermeier M, Jervis PJ, Cox LR, Graham SP, et al. The bovine CD1D gene has an unusual gene structure and is expressed but cannot present alpha-galactosylceramide with a C26 fatty acid. Int Immunol. 2013; 25(2):91–8. doi: 10.1093/intimm/dxs092 PMID: 22968995
14. Wang J, Guillaume J, Pauwels N, Van Calenbergh S, Van Rhijn I, Zajonc DM. Crystal structures of bovine CD1d reveal altered aGalCer presentation and a restricted A' pocket unable to bind very long-chain lipids. PloS one. 2012; 7(10):1–10.
15. Van Rhijn I, Nguyen TK, Michel A, Cooper D, Govaerts M, Cheng TY, et al. Low cross-reactivity of T-cell responses against lipids from Mycobacterium bovis and M. avium paratuberculosis during natural infection. Eur J Immunol. 2009; 39(11):3031–41. doi: 10.1002/eji.200939619 PMID: 19688747

16. Howard CJ, Sopp P, Parsons KR, Bembridge GP, Hall G. A new bovine leukocyte antigen cluster comprising two monoclonal antibodies, CC43 and CC118, possibly related to CD1. Vet Immunol Immunopathol. 1993; 39(1–3):69–76. PMID: 8116222

17. Howard CJ, Sopp P, Bembridge G, Young J, Parsons KR. Comparison of CD1 monoclonal antibodies on bovine cells and tissues. Vet Immunol Immunopathol. 1993; 39(1–3):77–83. PMID: 8116222

18. Howard CJ, Naessens J. Summary of workshop findings for cattle (tables 1 and 2). Vet Immunol Immunopathol. 1993; 39(1–3):25–47. PMID: 8116222

19. Parsons KR, MacHugh ND. Individual antigens of cattle. Bovine CD1 (BoCD1). Vet Immunol Immunopathol. 1991; 27(1–3):37–41. PMID: 2021059

20. Parsons KR, Howard CJ, Sopp P. Immunohistology of workshop monoclonal antibodies to the bovine homologue of CD1. Vet Immunol Immunopathol. 1991; 27(1–3):201–6. PMID: 2021059

21. O'Reilly KL, Splitter GA. Monoclonal antibodies to ovine SBU-T8 and SBU-T6 bind analogous molecules on bovine lymphocytes. Immunology. 1989; 67(2):176–80. PMID: 2787776

22. MacHugh ND, Bensaid A, Davis WC, Howard CJ, Parsons KR, Jones B, et al. Characterization of a bovine thymic differentiation antigen analogous to CD1 in the human. Scand J Immunol. 1988; 27 (5):541–7. PMID: 3287595

23. Hopkins J, Dutia BM, Rhind SM. Sheep CD1 genes and proteins. Vet Immunol Immunopathol. 2000; 73(1):3–14. PMID: 10678394

24. Rhind SM, Dutia BM, Howard CJ, Hopkins J. Discrimination of two subsets of CD1 molecules in the sheep. Vet Immunol Immunopathol. 1996; 52(4):265–70. PMID: 8896214

25. Van Rhijn I, Rutten VP, Charleston B, Smits M, van Eden W, Koets AP. Massive, sustained {gamma} {delta} T cell migration from the bovine skin in vivo. J Leukoc Biol. 2007; 81(4):968–73. PMID: 17234682

26. Hope JC, Whelan AO, Hewinson RG, Vordermeier M, Howard CJ. Maturation of bovine dendritic cells by lipopeptides. Vet Immunol Immunopathol. 2003; 95(1–2):21–31. PMID: 12969638

27. Im JS, Yu KO, Illarionov PA, LeClair KP, Storey JR, Kennedy MW, et al. Direct measurement of antigen binding properties of CD1 proteins using fluorescent lipid probes. JBiolChem. 2004; 279(1):299–310. PMID: 14551186

28. Dutia BM, Hopkins J. Analysis of the CD1 cluster in sheep. Vet Immunol Immunopathol. 1991; 27(1-–3):189–94. PMID: 2021059

29. Rhind SM, Hopkins J, Dutia BM. Amino-terminal sequencing of sheep CD1 antigens and identification of a sheep CD1D gene. Immunogenetics. 1999; 49(3):225–30. PMID: 9914336

30. Mackay CR, Maddox JF, Gogolin-Ewens KJ, Brandon MR. Characterization of two sheep lymphocyte differentiation antigens, SBU-T1 and SBU-T6. Immunology. 1985; 55(4):729–37. PMID: 3894224
